# Airborne Sound Power Levels and Spectra of Noise Sources in Port Areas

**DOI:** 10.3390/ijerph191710996

**Published:** 2022-09-02

**Authors:** Samuele Schiavoni, Francesco D’Alessandro, Davide Borelli, Luca Fredianelli, Tomaso Gaggero, Corrado Schenone, Giorgio Baldinelli

**Affiliations:** 1 Metexis s.r.l., Via Bartolomeo Grazioli 91, 06132 Perugia, Italy; 2 Italian National Research Council—Institute on Atmospheric Pollution at Italian Ministry of Ecological Transition, Via Cristoforo Colombo 44, 00147 Rome, Italy; 3 Department of Mechanical, Energy, Management and Transport Engineering, Division of Thermal Energy and Environmental Conditioning, University of Genoa, Via all’Opera Pia 15/A, 16145 Genoa, Italy; 4 Institute of Chemical and Physical Processes of National Research Council, Via G. Moruzzi 1, 56124 Pisa, Italy; 5 Department of Telecommunications, Electrical and Electronics Engineering and Naval Architecture, University of Genoa, Via Montallegro 1, 16145 Genoa, Italy; 6 CIRIAF—Inter University Research Centre for Environment and Pollution “Mauro Felli”, Via G. Duranti 67, 06125 Perugia, Italy

**Keywords:** port noise, noise sources, noise mapping, noise mitigations, noise modeling, ship noise, sustainable management, noise exposure prevention, noise measurements, research projects

## Abstract

Airborne port noise has historically suffered from a lack of regulatory assessment compared to other transport infrastructures. This has led to several complaints from citizens living in the urban areas surrounding ports, which is a very common situation, especially in countries facing the Mediterranean sea. Only in relatively recent years has an effort been made to improve this situation, which has resulted in a call for and financing of numerous international cooperation research projects, within the framework of programs such as EU FP7, H2020, ENPI-CBC MED, LIFE, and INTERREG. These projects dealt with issues and aspects of port noise, which is an intrinsically tangled problem, since several authorities and companies operate within the borders of ports, and several different noise sources are present at the same time. In addition, ship classification societies have recently recognized the problem and nowadays are developing procedures and voluntary notations to assess the airborne noise emission from marine vessels. The present work summarizes the recent results of research regarding port noise sources in order to provide a comprehensive database of sources that can be easily used, for example, as an input to the noise mapping phase, and can subsequently prevent citizens’ exposure to noise.

## 1. Introduction

Nowadays, the world economy is globally interconnected, which means that every year, larger amounts of goods need to be transported between countries and continents in a safe, efficient and competitive way. Since the second half of the twentieth century, when intermodal container shipping was invented in the U.S., this method of freight transportation has gained more and more importance, leading to the creation of large ports all over the world, with Asia leading the chart with the largest container ports. Over 80% by volume of the international goods traded are carried by sea, and the percentage is even higher for most developing countries. The COVID-19 pandemic affected maritime transport but the effects were lower than expected [1], even if, in February 2022, more than eleven percent of the global container ship capacity was unused, and the average shipping delay has also increased due to port congestion [2].

In addition to the maritime traffic generated by container shipping, passenger ships, and especially the leisure cruise industry, have experienced an annual passenger growth rate of 6.6% from 1990 to 2019. In this case, the effect of the pandemic was definitely greater, with almost one year of a complete stop on passenger cruises. On the other hand, the pandemic also accelerated the retirement of many ships leading to more modern and environmentally friendly fleets [3].

In light of the above, ports are crucial elements for the global market, but they may generate severe negative impacts, mostly related to the environment, land use and traffic congestion. The main negative environmental impacts are due to the emission of noise, odors and volatile organic substances, and to the pollution of water and soil by oil chemicals, hull paint and other hazardous materials [4]. Furthermore, most of these negative impacts are localized, taking place close to the port (in terms of noise and dust) and in the urban area (for air emissions, water quality, congestion and land use) [5]. In several cases, ports are located in close proximity to urbanized areas and they may even be bounded by or include environmentally protected areas [6]. Thus, it is evident how global needs and benefits produce local negative impacts.

From the noise point of view, ports are complex infrastructures if compared to other transport infrastructures (roads, railways, airports) and logistic nodes. The possible sources of noise that can be found in a port range from ships in transit to stationary ships, generators, maneuvering equipment, cranes, machinery and ventilation systems, but also moving vehicles and trains. Fredianelli et al. [7] made a comprehensive list of noise sources that can be found in a port area, which is reported in Section 2 of the present paper.

The great number of different sources of noise are dynamically distributed in space and time in a relatively large area, which is usually characterized by unsteady behavior or tonal components. The result is a sound environment with an extraordinarily variable temporal and spectral structure, where single sound sources are difficult to isolate. Furthermore, many of them are characterized by prominent low frequency components, between 20 Hz and 200 Hz, which can travel long distances with limited attenuation and are hardly insulated from building walls [8].

For all of the above-mentioned reasons, ports are complex environments for professionals to deal with, also because specific noise-related rules and regulations for ports are often missing, both at the international and national level. For instance, the European Directive 2002/49/EC on the Assessment and Management of Environmental Noise (END) is focused on two main categories of noise sources: transport infrastructures and industrial sites. Even if the END specifies that industrial (port) areas near large agglomerations must be included in noise maps, it gives no specific indications on how to draw their noise maps. As a consequence, noise nuisance in port areas is usually addressed by considering the ports as a single noise source, similar to an industrial area, with evident underestimation of the issues. With the absence of a uniform approach or guidelines, when problems arise regarding citizens affected by port noise [9], it is generally addressed at a local level by following different approaches that only tend to respond to complaints. In Italy, as a matter of example, even if specific decrees that regulate the noise produced by roads, railways, airports and industries exist, a national decree for the regulation of noise generated by port activities is still missing, although it is required by national law.

Ports, as noise sources, and industrial noise in general, are neglected by the World Health Organization too, with its 2019 environmental noise guidelines for the European region [10], providing policy makers with recommendations for protecting human health from exposure to environmental noise originating from transportation (road traffic, railway and aircraft), wind turbine noise and leisure noise. As was observed by Bernardini et al. [11], unlike the broad literature on transportation noise and industrial noise and the variety of mitigation measures for those sources, the scientific community paid very little attention to analyzing and tackling the noise produced by ports and the effects of the exposure on the surrounding population. Furthermore, the majority of scientific research regarding the noise generated from maritime transport is focused on studying onboard vessel noise, its interference with animal life or oceanic ambient noise. In this field of research, in fact, underwater noise is investigated more than airborne noise.

Evidently, planning or managing noise in port areas can be an overwhelming task for even the most skilled professional, especially when it comes to simulating noise propagation and evaluating noise levels at the receivers’ location. The main problem is nearly always in defining the noise sources that are located inside the port area, even if the definition of the port boundary can be a problem on its own [12], in addition to where and for how long the sources are operating, and most importantly, their characterization in terms of directivity and sound power.

The present study reviews the current scientific literature, technical report and other databases with the aim of collecting the sound power levels of the different noise sources acting in port areas. The comprehensive list that is created would be very useful for professionals and technicians as input data for simulation and the mapping phase needed in the noise management toward citizens’ health care. Moreover, the work also acts as a further starting point for driving future work into a harmonized approach of study regarding port noise.

The following sections of this paper are structured as follows: Section 2 summarizes the recently published guidelines for noise mapping of port areas, Section 3 collects the noise emission data of major port sources, divided according to the most used typology: transtainer, reach stacker, straddle carrier, gantry cranes, reefer, moored ships, Ro-Ro and Ro-Pax ramps, forklifts, and seagoing ships. Section 4 provides the conclusions to the work.

## 2. Available Source Characterization Guidelines for Noise Mapping of Port Areas

A joint product of the Interreg European projects REPORT, MON ACUMEN, DECIBEL and RUMBLE was the development of source characterization guidelines for noise mapping of port areas. As described by Fredianelli, et al. [13], the aim of the work was to present specific measurement procedures for the assessment of noise emissions of the many sources acting in ports, according to the five macro-categories and further sub-categories previously proposed in another work [7]. The procedure was set in order to provide technicians and stakeholders with a unified methodology that allows the retrieval of the inputs for noise mapping software. The guideline expectations were to boost sector studies and start providing a common approach to acoustic mapping of ports that will allow a proper comparison of population exposure levels in the future.

The categories for which a specific procedure was reported are:

- Road:
  - Internal traffic;
  - Port-related external traffic;
  - External traffic not generated by the port.
- Railways:
  - Internal traffic;
  - Port-related external traffic;
  - External traffic not generated by the port.
- Ships:
  - Sailing at a reduced speed approaching the quay;
  - Moored in stationary conditions;
  - Mooring operations;
  - Moored during loading/unloading operations (without auxiliary machinery).
- Port and industrial:
  - Fixed sources;
  - Mobile sources;
  - Area sources.

In fact, all the sources falling into the previously mentioned categories have peculiarities that led to a different noise emission with respect to the others, which obviously translates into the need for a specific measurement procedure. Only minor adaptations to port environment scenarios were proposed for roads and railways, which are well characterized sources with CNOSSOS-EU as a proper model [14]. The modifications considered the high percentage of heavy and freight vehicles with respect to passenger vehicles in the port infrastructure, with the annexed average reduction in speed.

All of the choices made in defining the measurement procedures for ships, port and industrial sources were made assuming the lower interference with port and ship operations and the impossibility to measure onboard. In addition, the measurement procedures do not need the collaboration from both ships or terminal owners, in addition to switching the machinery off/on on request to reduce the background noise. Furthermore, the use of cranes or cherry pickers to reach a higher measurement position was discarded for the sake of simplicity and economy. A simplification made in the work was considering ships as the emitting noise in a symmetric way with respect to its vertical longitudinal symmetry plane, even if this assumption is not true for some vessels. This allows technicians to perform characterization measurements on only one side and then avoiding renting boats to reach the side facing the sea of the moored ship.

Port and industrial categories include the same machinery and vehicles used in port activities or for the industries in the port area, but a different classification is needed for better identifying the legal responsibility of limit exceedances. As both categories are very wide, a further subdivision was carried out in the moving or fixed source, or even the area source when details are not important. Pumps, generators, ventilators, air conditioners, machinery of any type, fixed cranes, conveyor belts and refrigerated containers are some of the fixed sources that can be found in a port environment. Instead, straddle carriers, frontlifts, contstackers, forklifts, transtainers, cranes, dock tractors and other cargo handling units are the mobile sources. The measurement procedure for a mobile source is different according to its operation phase (transit, handling, loading/unloading operations), which should be all properly characterized.

All of the noise measurements must be performed with a class I [15] sound level meter. The sound power level (L_W_) of the investigated source is calculated from the sound pressure levels (L_p_) obtained using Equation 1, possibly separated in third octave bands.

L_W_ = L_p_ + 10·log[Q/(4πr^2^)], (1)

where Q is the directivity factor and r is the distance to sound source.

The resulting sound power level L_W_ is the information needed by the models to simulate the noise in port areas.

For the purpose of the present work, however, the guidelines represent the first attempt to drive the collection of sound emitted by port sources with the intent of stimulating the scientific community to create an accessible and comprehensive database.

## 3. Collection of Noise Emission Data of Major Port Sources

The following reports a selection of the most solid and consistent data concerning sound emitted by sources located inside port areas, retrieved from the scientific and technical literature as well as from the reports of the European projects cited in the previous paragraphs; data are reported as sound power level L_W_ for each source and expressed in dB or dB(A) for point sources, in dB/m or dB(A)/m for linear sources and in dB/m^2^ or dB(A)/m^2^ for area sources.

The retrieved information regarding the sound power levels of noise sources operating in port areas are mainly gathered from:

- The outcomes of the REPORT project [16] for transtainer, reach stacker, reefer and gantry cranes;
- The paper “Noise evaluation of sound sources related to port activities” [17] for Ro-Ro and Ro-Pax vessels, rubber-tired gantries, straddle carriers, reach stackers, reefers and the ramp noise, realized within the activity of the EU funded EFFORTS project [18];
- The paper “Container Terminals and Noise” for container ships, straddle carriers and tractors [19];
- The report “Technical noise investigations at Hamburg City cruise terminals” of the INTERREG Green Cruise Port for moored cruise ships, reefers and forklifts [20];
- The outcomes of the NEPTUNES project for ships and other sources [21];
- The paper “Noise emission Ro-Ro terminals” for Ro-Ro moored ships [22];
- The paper “Airborne noise emissions from ships: Experimental characterization of the source and propagation over land” for container ships [23];
- The paper “Evaluation and control of cruise ships noise in urban areas” for cruise ships [24];
- The report “Noise from ships in ports” for moored ships and reefers [25];
- The report of the Lloyd’s Register regarding how the noise emissions of a moored ship have to be modelled [26];
- The report “Assessment of the acoustic benefit of the power supply to ships moored in ports (cold ironing)” [27] and the related paper presented at the Euronoise 2018 Conference [28];
- The paper “Pass-by Characterization of Noise Emitted by Different Categories of Seagoing Ships in Ports” [29];
- The outcomes of the FP7 SILENV project for the moored ships ([9,30,31,32,33]);
- ISPRA (Italian National Institute of Environmental Protection and Research) data based on the FP7 SILENV project [34,35].

A summary of the standards used in the acoustic measurements carried out to define the sound power level of the port noise sources described in the documents above can be found in Appendix A.

### 3.1. Transtainer

**Figure 1:**
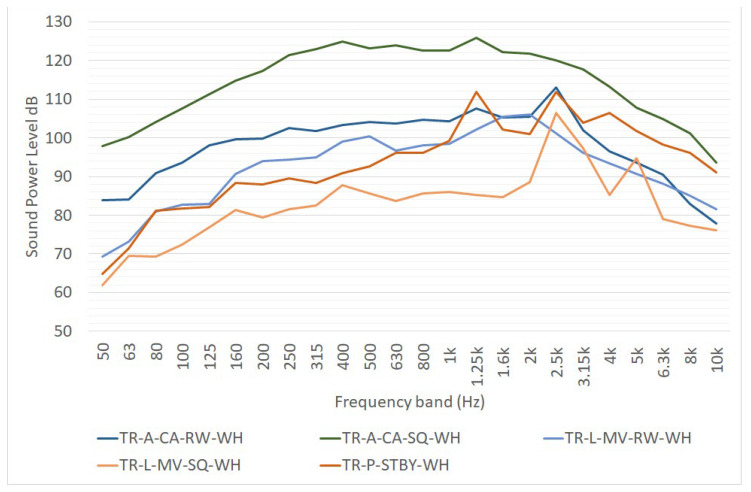
One-third octave sound power level spectra of a transtainer, as reported in [16]. L_W_ is expressed in dB for point sources, in dB/m for linear sources and in dB/m^2^ for area sources.

The REPORT project [16] gives the one-third octave band’s sound power level spectra of a transtainer on standby, in movement with the alarm signal horn functioning and in full activity. The standby emission is represented by a point source; the moving transtainer with the alarm signal being modelled as a linear source. The activity of the transtainer is made by all the movements it makes to take, move and place containers in port areas. Data collected from noise measurement in the REPORT project evidence that transtainer emissions on the railway side are different from the ones on the square side. Figure 1 reports the sound power emission spectra of a transtainer, as calculated in the REPORT project:

- TR-A-CA-RW-WH considering an equivalent area source, device performing a complete activity, measurement focused on the railway side;
- TR-A-CA-SQ-WH, same as the previous item, but focused on the square side;
- TR-L-MV-RW-WH considering a linear noise source representing the movement of the transtainer on the rail, measurement focused on the railway side;
- TR-L-MV-SQ-WH considering a linear noise source representing the movement of the transtainer on the rail, measurement focused on the square side;
- TR-P-STBY-WH considering a point noise source representing the transtainer on standby. The point noise source can be used to model the emission of the whole device.

**Figure 2:**
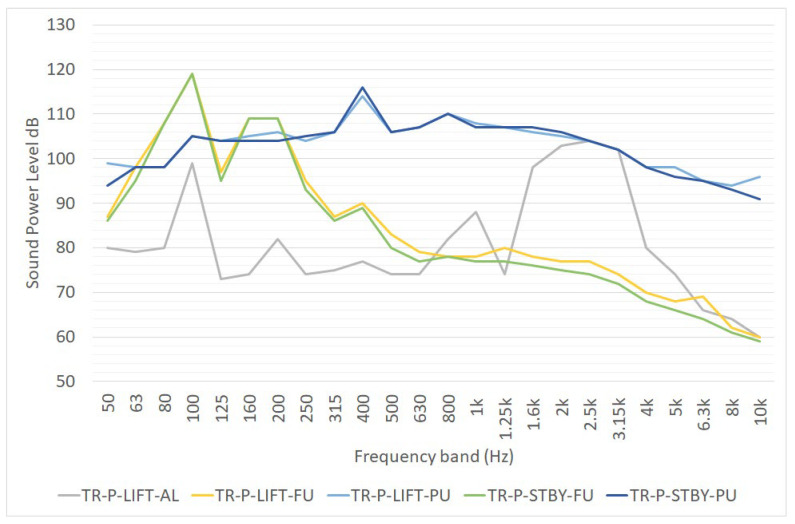
One-third octave sound power level spectra of a transtainer, as reported in [17,18].

Another characterization of this noise source was performed within the activities of the EFFORTS project [17,18]. The noise emission characteristics of the device was defined by three different point sources representing the power unit, the exhaust pipe (20 m above the ground) and the alarm signal. Figure 2 reports the following data:

- TR-P-LIFT-AL is the sound power level of a point noise source representing the noise emission of the alarm signal when the transtainer is performing an operation of lifting or picking up containers;
- TR-P-LIFT-FU is the sound power level of a point noise source representing the noise emission of the funnel when the transtainer is performing an operation of lifting or picking up containers;
- TR-P-LIFT-PU is the sound power level of a point noise source representing the noise emission of the power unit when the transtainer is performing an operation of lifting or picking up containers;
- TR-P-STBY-FU is the sound power level of a point noise source representing the noise emission of the funnel when the transtainer is on standby (idling);
- TR-P-STBY-PU is the sound power level of a point noise source representing the noise emission of the power unit when the transtainer is on standby (idling).

Moreover, the noise emission of the equipment was obtained through measurement under idling and lifting conditions.

Sound power data from the REPORT Project [16] represent the noise source as a single source, so it can be handled more easily than those of the EFFORT Project [18]. Nevertheless, it is worth nothing that noise measurements used for calculating the sound power level in [18] were carried out in compliance with ISO 3744. There is no information about the standard of the noise measurement carried out in [16].

### 3.2. Reach Stacker

**Figure 3:**
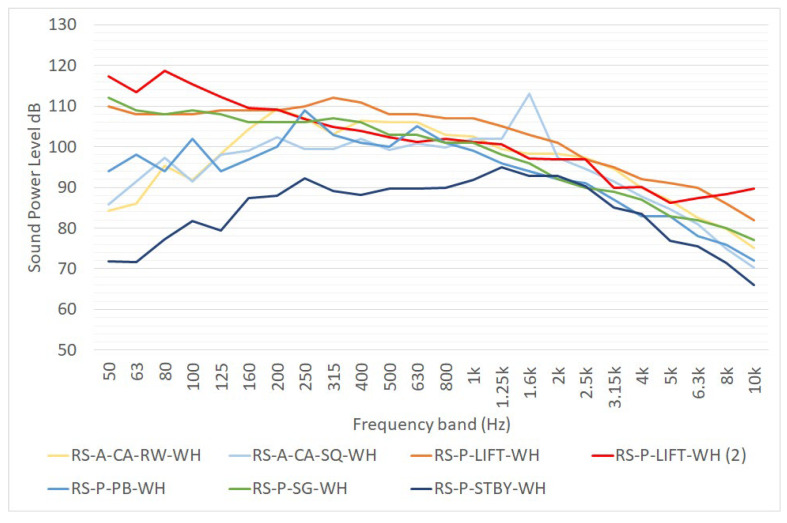
One-third octave sound power level spectra of a reach stacker, as reported in [16,17,18,36]. L_W_ is expressed in dB for point sources, in dB/m for linear sources and in dB/m^2^ for area sources. RS-A-CA-RW-WH, RS-A-CA-SQ-WH and RS-P-STBY-WH data are taken from [16], RS-P-LIFT-WH, RS-P-PB-WH and RS-P-SG-WH from [18], RS-P-LIFT-WH (2) from [36].

The REPORT [16] and the EFFORTS projects [17,18] estimated the noise emitted by reach stackers. Figure 3 reports the following data:

- RS-A-CA-RW-WH and RS-A-CA-SQ-WH are the sound power levels of two equivalent areal noise sources representing the noise emission of two different reach stackers performing a complete activity, as estimated in [16];
- RS-P-LIFT-WH and RS-P-LIFT-WH (2) are the sound power levels of point noise sources representing the noise emission of the reach stacker when it is performing an operation of lifting or picking up containers, respectively, estimated by [18,36]. The latter was derived from the global sound power level (L_w,sum_), considering a pink noise source;
- RS-P-PB-WH is the sound power level of a point noise source representing the noise emission of the device pass-by [18];
- RS-P-SG-WH is the sound power level of a point noise source representing the noise emission of the device performing an operation of setting containers to the ground [18];
- RS-P-STBY-WH is the sound power level of a point noise source representing the noise emission of the device in standby mode (idling) [16];

### 3.3. Straddle Carrier

**Figure 4:**
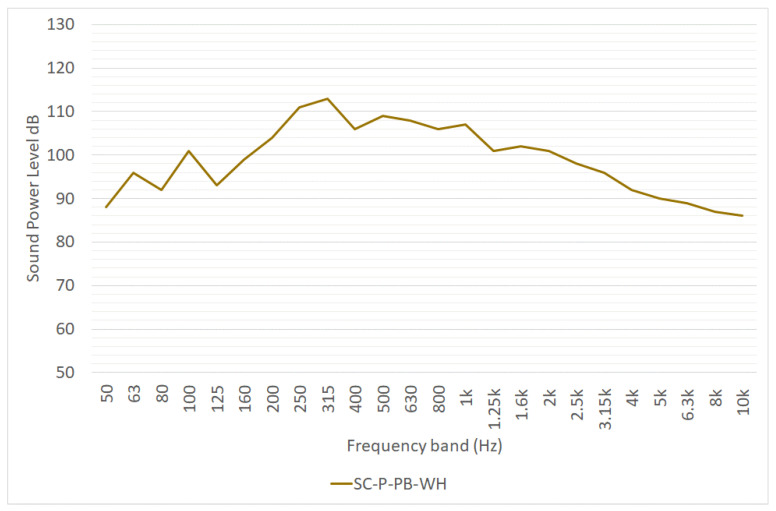
One-third octave sound power level spectrum of a straddle carrier pass-by, as reported in [17]. L_W_ is expressed in dB/m for linear sources.

The noise emission of a straddle carrier has been determined using the pass-by method by [17] within the activity of the EFFORTS project: the sound power level of the equivalent point noise source SC-P-PB-WA is reported in Figure 4 and in Table 1. The overall sound power level was reported in the deliverable 2.4.3 of the project [18]. Another remarkable study investigating noise emission from straddle carriers were carried out by Witte [19]. This study does not provide spectral information, but only reports the overall sound power level in dB(A), without specifying how these data were calculated (Table 1).

### 3.4. Gantry Cranes

**Figure 5:**
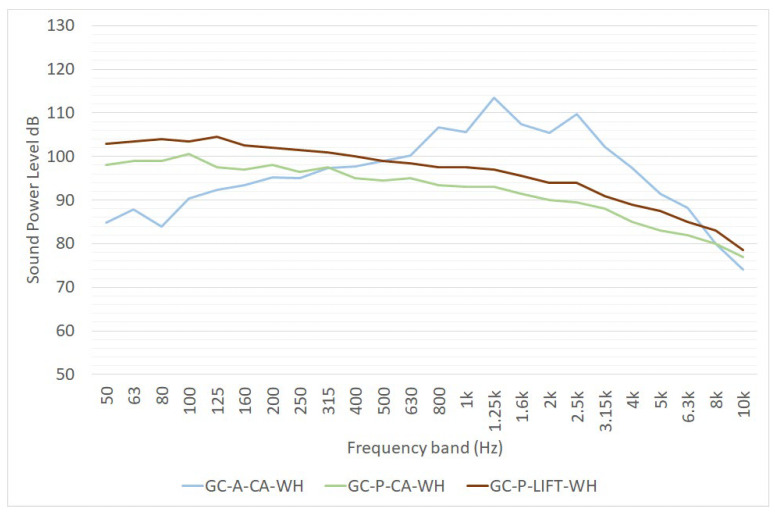
One-third octave sound power level spectra of gantry cranes, as reported in [16,17,18]. L_W_ is expressed in dB for point sources, in dB/m for linear sources and in dB/m^2^ for area sources. GA-P-CA-WH and GA-P-LIFT-WH data are taken from [17,18], GC-A-CA-WH from [16].

The REPORT [16] and the EFFORTS projects [17,18] estimated the sound power level spectra of gantry cranes. Figure 5 shows the following data:

- GC-A-CA-WH is the sound power level of two equivalent areal noise sources representing noise emission due to the complete activity of a gantry cranes, as estimated in [16];
- GA-P-CA-WH and GA-P-LIFT-WH are the sound power level spectra of point noise sources representing the noise emission of the complete activity and of the lifting operation alone, respectively. These data were estimated in [17,18].

These data were obtained by the analysis of noise measurements. Witte provided a rough estimation of sound power level equal to 100 dB(A) for gantry cranes in [19], without providing further information relating to how the data were obtained (noise measurements, databases, etc.).

### 3.5. Reefer

Several data are available relating to the assessment of the noise emission of reefers, i.e., refrigerated containers. The characterization of this kind of equipment is easier in comparison to other port noise sources since it is a regular container with an HVAC unit devoted to maintaining an adequate temperature inside.

**Figure 6:**
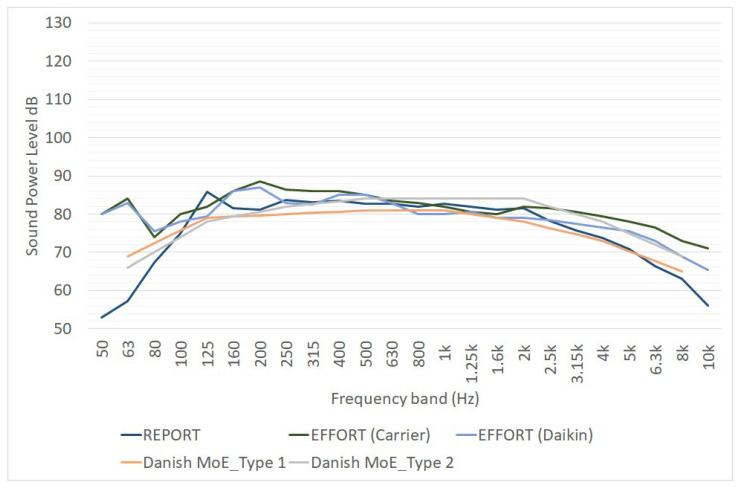
Sound power level spectra of reefers, as defined by REPORT Project [16], EFFORT Project [18] and a report of the Danish Ministry of Environment [25]. L_W_ is expressed in dB for point sources, in dB/m for linear sources and in dB/m^2^ for area sources.

Noise measurements were used to characterized the one-third octave acoustic emission of reefers in both the REPORT project [16] and the EFFORTS project [17,18] (Figure 6); the figure also contains octave band data taken from a 2010 report of the Danish Ministry of Environment [25].

Other noise emission assessments of reefers based on measurements have been carried out by the NEPTUNES project [21] and by the Interreg-funded project Green Cruise Port [20] (see Table 2).

The differences observed in terms of noise emission are probably caused by the different models of the cooling units installed in each device. It is worth nothing that only data in REPORT project give additional information regarding how the noise measurements were carried out.

### 3.6. Moored Ships

The best practice guide “Mitigation of Noise from Ships at Berth” of the NEPTUNES project [21] suggests that ships may be divided into six different classes:

- Container ships;
- Cruise ships;
- Tankers;
- Ro-Ro and Ro-Pax;
- Bulk Carriers;
- General cargo/ service ships.

Their noise emissions are caused by:

- The funnel outlet(s) of the auxiliary engine(s), all ship types;
- The opening of the engine room ventilation inlet(s) and outlet(s), all ship types;
- The opening of the cargo hold ventilation and air conditioning inlet(s) and outlet(s), all ship types;
- The opening of the ventilation and air-conditioning of passenger rooms (cruise ship and Ro-Pax);
- Further relevant ventilation openings;
- Pumps on deck (tankers).

The project gives some indicative values of the noise emission of a moored ship; they are reported in Table 3. These data cannot be used for noise assessment studies because they oversimplify the complexity of the acoustic emission of a moored ship. However, they can give an indication regarding the impact of the noise emission of a ship at berth without a cold ironing solution.

A more detailed study regarding the assessment of the noise emission of a ship at berth was carried out by the Danish Ministry of the Environment [25]. The study reports the sound power level of some types of diesel generators without silencers (Table 4) and ventilation fans (Table 5) used in vessels, collected from the producers of these components. The data evidences that the anti-noise treatment of the engine is crucial to reducing the noise impact of a moored ship.

It is worth noting that a good approximation of the sound power level of a fan is given by Equation (2), reported in [37]:

L_W_ = L_w_^*^ + 10·q_v_ + 20·Δp_v_, (2)

where:

- L_W_^*^ can be assumed to be 25–30 dB for radial ventilators and 25–35 dB for axial ventilators;
- q_v_ is the volume flow in m^3^/h;
- Δp_v_ is the fan total pressure difference in Pa.

**Figure 7:**
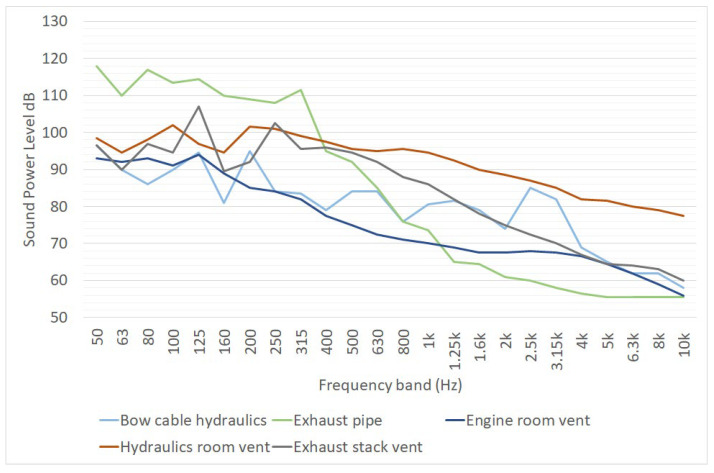
One-third octave sound power spectra of Ro-Ro vessel noise sources, as reported in [17]. L_W_ is expressed in dB for point sources, in dB/m for linear sources and in dB/m^2^ for area sources.

The noise emission of a Ro-Ro vessel at berth was also studied in the EFFORTS project ([17,18]); the sound power level spectra were obtained from noise measurements performed in the ports of Turku and Dublin (Figure 7). The overall sound power levels of these sources provided by the EFFORT project have been compared with similar data reported in a 2018 technical report delivered by Tecnalia [27] (Table 6). Both studies considered a Ro-Ro ship at berth; it is worth noting that the noise source “Auxiliary engine” in [27] likely groups together some of the sources considered in [17].

An approximate and quick assessment of the noise emission of container ships was carried out by Witte [19]: the relationship (Equation (3) between the deadweight tonnage (DWT) and the A-weighted sound power level of a container ship can be expressed as follows:

L_W,A_ = 55.4 + 12.2·DWT (3)

The equation was obtained by noise measurements on 65 ships. The use of this data to characterize the noise emission of a container ship has some drawbacks:

- The container ship is modelled through a single point noise source. This approach may lead to relevant errors in the assessment of noise impacts, in particular for receivers located close to the docks;
- The author does not provide detailed information about how the noise measurements were carried out and processed.

An accurate characterization of a container ship has been performed in [23], considering a detailed digital model of the vessel and the noise emission spectra of each source. The emission spectra were obtained by tailored measurements. In order to validate the noise model, horizontal and vertical grids of noise measurement were performed. The outcomes proved that “a limited number of dedicated onsite measurements together with adaptations of the code to the specific case allowed us to obtain an effective model for the ship”.

**Figure 8:**
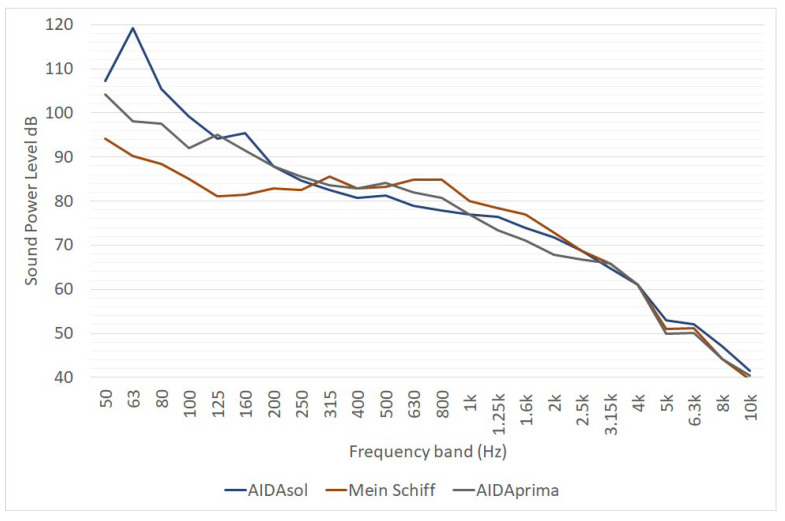
One-third octave sound power spectra of cruise ship funnel of three vessels, as reported in [20]. L_W_ is expressed in dB for point sources, in dB/m for linear sources and in dB/m^2^ for area sources.

The report of the Interreg-funded project Green Cruise Port [20] provides much information. Noise emission data of cruise ships were obtained from noise measurements considering separate exhaust gas outlets (Figure 8) and the ventilation openings of three vessels: AIDAsol (Length equal to 253/Width 38 m), AIDAprima (300/48 m) and Mein Schiff 3 (294/39 m).

Concerning the exhaust gas outlet group, the three vessels have similar noise emission spectra for frequencies higher than 200 Hz. Under this threshold, the noise emission of the AIDAsol (the smaller one) is more relevant than for the other two ships. The equivalent sound pressure level L_w,A_ is of 102 dB(A) for AIDAsol, 100 dB(A) for AIDAprima and 98 dB(A) for Mein Schiff 3. Noise measurements for the characterization of exhaust gas outlets were carried out in compliance with DIN 45635-47.

**Figure 9:**
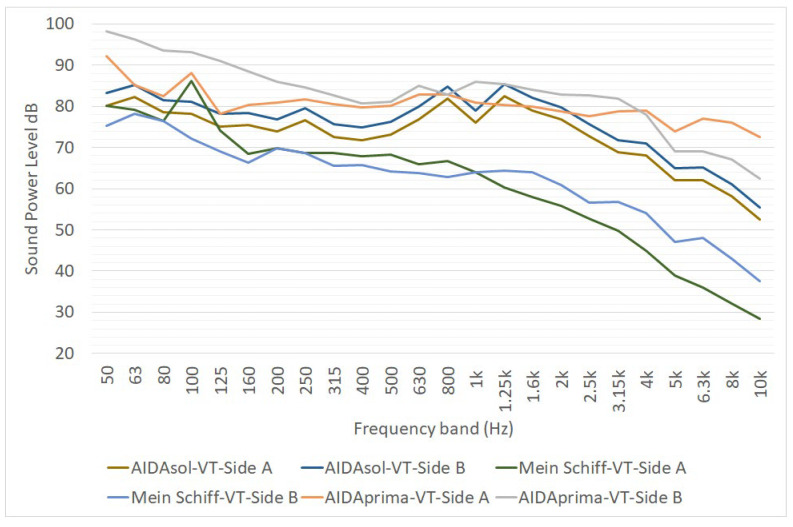
One-third octave sound power spectra of ventilation openings on the two sides of three vessels, as reported in [20]. L_W_ is expressed in dB for point sources, in dB/m for linear sources and in dB/m^2^ for area sources.

Concerning ventilation openings, noise measurements carried out on the three vessels evidenced that they may have a tonal (peak at 100 Hz) or a broadband character. The elaboration of the noise measurements performed in the Green Cruise Port project evidenced that the noise emission from the two side of the same cruise ship can be substantially different (Figure 9). Noise measurements carried out for the characterization of ventilation openings were carried out in compliance with DIN EN ISO 3746 [38].

In 2009, Witte carried out a noise measurement campaign to define a relationship between the loading capacity and noise emission of Ro-Ro ships, such as the one defined for container ships. However, in this case, the author did not find any relationship between the two parameters, as reported in [22].

**Figure 10:**
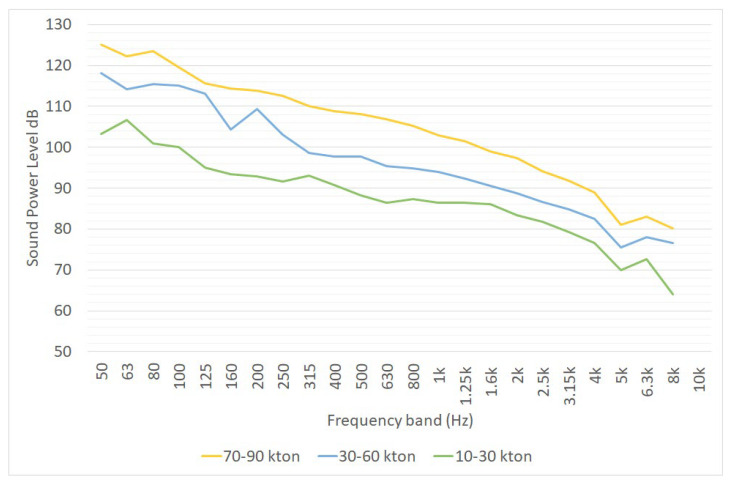
One-third octave sound power spectra of cruise ships divided into big (70 to 90 ktons), medium (30 to 60 ktons) and small size (10 to 30 ktons), as reported in [24]. L_W_ is expressed in dB for point sources, in dB/m for linear sources and in dB/m^2^ for area sources.

A 2013 paper by Di Bella and Remigi reports the one-third sound power spectra of several typologies of cruise ships; they were evaluated as single-point noise sources and their sound power level was estimated from the elaboration of in-field measurements performed in compliance with several ISO standards. The spectra of these sources are reported in Figure 10 [24]. Nevertheless, the outcomes of some studies evidence that the characterization of a moored ship as a single-point noise source seems to be an excessive approximation ([33,39]).

A recent report on the noise emission of moored ships was issued by Tecnalia in 2018 [27]. The report was focused on evidencing the reduction of noise impacts thanks to cold ironing systems. In addition, the report contains useful indications about the noise emission of several kinds of moored ships based on noise measurements performed in Spanish ports in 2017; these noise data are reported in Table 7.

Even if it does not contain sound power data, it is worth considering the operational indications provided by the 2019 report of the Lloyd’s Register on moored ship noise modelling [26]. The document suggests that the noise modelling procedure should consider the screening, reflection and absorption procedure of the ship’s structure and should be performed at least in the 31.5–8000 Hz range. The inclusion of the following noise sources is recommended:

- Funnels and other exhaust stacks;
- Ventilation air intakes and exhaust;
- External fans;
- Hull radiated noise (if relevant);
- Cranes, pumps and any other equipment in operation.

They suggest considering the noise sources as single-point emitters, with the exception of large ventilation openings, which should be considered as surface noise source. The noise emission directivities of the noise sources have to be considered.

It may be of interest to mention the different approach used by Moro [40]; the noise emission of 290 meters and a 110,000 DWT cruise ship was modelled through software based on the beam method. The ship was defined through a 3D geometry model and all its noise sources were detected and characterized in terms of the sound power level. These emissions were defined using the procedures in compliance with the ISO 3744 standard. A comparison between the outcomes of this model with a noise measurement campaign was carried out and the outcomes showed an adequate agreement.

Finally, it is worth nothing that the FP7 SILENV project [30] defined two different methods to assess the noise emission of moored ships, as is reported in detail in the deliverable 5.2 of the project, “Noise and vibration label proposal” [31,32]. Each moored ship has to be modelled through a group of point, linear and area noise sources. Each one of these sources represents the relevant noise emitters of a moored ship such as funnels, intake and outlets of ventilation, and HVAC, etc.

### 3.7. Ro-Ro and Ro-Pax Ramp

The noise caused by the passage of vehicles on the Ro-Ro and Ro-Pax ramps can be relevant in a noise mapping project. Unfortunately, few works have been dedicated to its noise assessment. The EFFORTS project ([17,18]) allows us to give an estimation of these noise events in terms of sound power level spectra (Table 8). The assessment of noise emission was carried out through measurements on three ramps, one in Turku and two in Dublin. In the Turku assessment, the movement of goods was made through tractors; in the Dublin assessments, the ramps were used directly by trucks. Data reported in Table 8 shows that the noise emission data obtained from this study were subjected to a high degree of variability.

**Figure 11:**
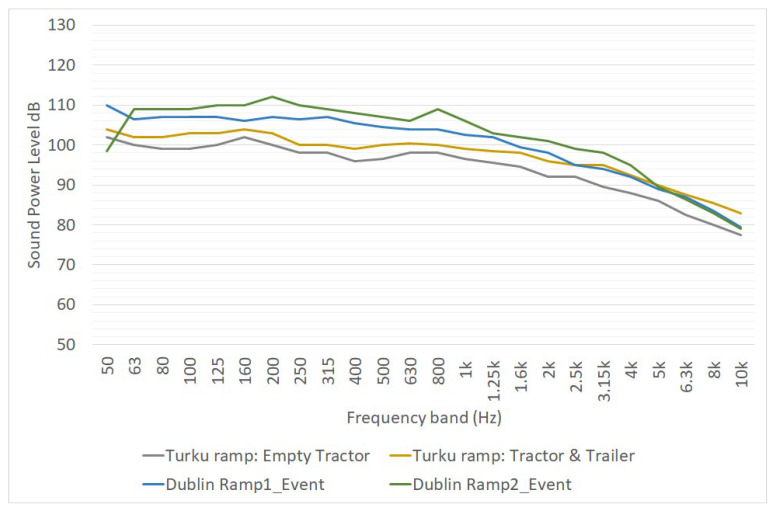
One-third octave sound power spectra of Ro-Ro ramps, as reported in deliverable 2.4.3 of [18]. L_W_ is expressed in dB for point sources.

The spectra of sound power levels from Table 8 are reported in Figure 11. It is worth noting that the spectra of the two ramps in Dublin are sensibly different; this is probably caused by the peculiarity of this noise source. Ramp noise emission is caused by the bumps between the ramp and the ground that happen when a track or a tractor passes over it. Each bump causes noise emissions that are considerably different from the others; there are a lot of factors influencing this (ground and ramp typology, tractors or truck velocity, weight of the tractors, etc.). In noise simulation activities, it is recommended to perform some devoted noise measurements on ramps similar to the ones to be modelled. Only if this is not possible, should this data reported in Figure 11 and Table 8 be used.

### 3.8. Forklifts

The Green Cruise Port project [20] provided an overview of the noise emission of forklifts in a port area (Table 9). However, the sound power level of these devices can be retrieved from many databases, because they are also used outside port areas and their manufacturers are obliged to explicitly declare this data according to the 2000/14/EC directive. For instance, the SoundPLAN 8.2 database includes spectra for several kind of forklift, both under idling and in working conditions [41].

### 3.9. Seagoing Ships

In the harbor context, ships can represent an important source of noise both when berthed and when under navigation or during maneuvers. Navigation in the harbor is always at a very low speed; nevertheless, all the noise sources present during navigation are active such as air inlets and outlets, machinery noise, funnel noise, cargo treatment, etc. For now, measurement standards are available for vessels in inland waterways [44] and for small pleasure crafts [45], while no specific standard for seagoing ships is available. As a consequence, the scientific community has followed different approaches in measuring and reporting their results [46,47]. In [48], eight transits of two ferries (A, B), considering arriving and leaving with a source–receiver distance of about 170 m, were measured. Results are reported in Table 10 in terms of A-weighted SEL.

No spectra were reported in the original work for a single ship, but a spectrogram of a single ship passage highlights the presence of string noise components at lower frequencies.

In [49], several measurements were carried out of a ferry during maneuvers in the port of Naples. The sound level meters were placed in two different locations: one on the short berth side and one on the long berth side. No spectral results were provided.

Fredianelli, et al. [29] performed the most complete effort to define the noise emission of different categories of seagoing ships by means of long-term pass-by measurements in the port of Livorno. The average sound power levels and spectra of different categories of big ships are reported in Table 11. Among the categories are: Ro-Ro, container, oil tanker, chemical tanker and ferry. The values for ferries are taken for a subsequent study by the same authors [50], focused only on ferries and the effects of parameters, such as ship speed and draught, and the distance from the microphone, have on the measured noise. Table 11 also includes the sound emissions of small and medium vessels performed by Bernardini, et al. in [11]. According to the paper, small vessels are meant to be small motorboats, sailing boats, and rigid-hulled inflatable boats, while medium vessels are small and mid-sized fishing boats, fireboats, and public security boats. Data reported in Table 11 for small and medium vessels are those reported in the original paper as “medium speed”, 14.4–19.2 km/h (7.8–10.4 kn) for small vessels and 9.2–10.7 km/h (5.0–5.7 kn) for medium vessels.

Results consistently show the typical presence of strong noise components at lower frequencies for each ship type.

### 3.10. Other Sources

Port areas can also be occupied by more common noise sources such as roads, rail, industries, power plants, waste treatment plants, etc. The assessment of the noise emission of these activities can be carried using the available CNOSSOS models [14]. Noise emission from parking can be evaluated using, for instance, the model developed by [51,52]. The assessment of the noise emitted from leisure activities is outside of the scope of this paper.

## 4. Conclusions

A comprehensive database of typical sound power levels and spectra of the various noise sources acting in ports was developed in the present paper. The sound power levels and spectra reported and summarized in the present work come from the research on both the scientific and technical literature, as well as from the deliverables and results of several European projects. These projects were developed on the framework of different programs (i.e., FP7, Interreg, ENPI CBC-MED, LIFE). Among them, the most important projects to be mentioned for having produced the higher amount of data are ANCHOR, REPORT and SILENV. As some of the authors participated in more than one of these projects, albeit with different aims of the funding programs and of the projects themselves, working in a sort of cluster of projects dealing with the same topic led to the development of knowledge on the topic that allowed the understanding of several different issues about port noise and the finding of solutions that are able to tackle them. One of the key issues was, in fact, the difficulty of developing environmental noise simulations of port noise leading to accurate and repeatable strategic noise maps and the subsequent noise action plans, as requested by the Environmental Noise Directive of the European community. In general, a scarcity of data was found for all sources considered. This may be due to a certain lack of standards and normative frameworks specific to these kinds of problems. In particular, the largest sources (such as transtainers and container ships) are those for which the lower amount of data is present. Moreover, it has to be underlined that sometimes the data sources avoid specifying whether the characterization measurements were made in compliance with a standard; this leads to a non-uniformity that makes difficult to compare results and to assess the reliability of the data presented. New guidelines aimed at tackling these issues were developed within the framework of the already cited REPORT project, recently ended, and it is foreseeable that these guidelines will be enacted in the future in order to have reliable, comparable and consistent measurements regarding port noise sources.

Such an organized dataset would be important for the present state of the scientific and technical literature as the complexity of noise produced by port infrastructure is high, with many different sources acting simultaneously. In recent years, port noise has been the object of some studies, but mostly those investigating only a singular type of noise source. Thus, a database comprising all of the spectral data retrievable was missing in the literature to the best of the authors’ knowledge and it would be beneficial for technicians who produce noise maps or for other scientists willing to further improve a topic that still deserves lot of attention.

The development of such a database will act as a base for developing reliable numerical simulations in order to comply with the evermore restrictive standards and normatives regarding environmental pollution and the sustainability of transportation infrastructure.

## Figures and Tables

**Table 1:** Sound power level of straddle carriers as reported in [18,19].

Straddle Carrier Activity	L_W_ (dB)	L_W_ (dB(A))
Pass-by [18]	119 ± 2	115 ± 2
Normal activity, power unit close to the ground [19]	/	108
Normal activity, power unit located at the top [19]	/	104

**Table 2:** Sound power level of reefers, as reported in [20,21].

Source	Working Condition	L_W_ (dB(A))
NEPTUNES Project [21], mitigation of noise from ships at berth	Normal activity	91–93
GREEN CRUISE PORT [20]	Normal activity	99

**Table 3:** Approximate sound power level of some port noise sources given by [21].

Source	L_W_ (dB(A))
Container ship	100–115
Ro-Ro ship	100–114
	(1)

**Table 4:** Unattenuated one-third octave sound power level in dB(A) of the different diesel engine exhausts [25].

Producer	Type	One-Third Octave Frequency Bands (dB(A))										Total (dB(A))
		16 Hz	31.5 Hz	63 Hz	125 Hz	250 Hz	500 Hz	1000 Hz	2000 Hz	4000 Hz	8000 Hz	
MAN B and W	L32/40	80	115	130	135	129	133	135	135	133	130	142
	V32/40	82	111	126	133	129	133	135	135	133	130	142
	L48/60B	88	119	124	126	129	133	135	135	133	130	141
	V48/60B	84	111	124	126	129	133	135	135	133	130	141
	L58/64	80	115	130	135	129	133	135	135	133	130	142
Wärtsilä	W26	-	122	132	135	131	125	124	118	112	102	138
	W32	-	107	115	127	130	129	127	121	109	-	135
	W38	-	101	119	122	127	131	134	129	126	118	138

**Table 5:** One-third octave sound power level in dB(A) of different ventilation fans used in vessels without anti-noise measures [25].

Fan Function	Volume Flow (m^3^/h)	One-Third Octave Frequency Bands (dB(A))								Total (dB(A))
		63 Hz	125 Hz	250 Hz	500 Hz	1000 Hz	2000 Hz	4000 Hz	8000 Hz	
Engine room fans	120,000	73	93	98	105	105	102	98	91	110
	70,000	68	84	100	104	106	103	99	93	110
	50,000	66	82	98	101	103	101	97	90	108
	33,000	64	79	96	99	101	99	94	88	106
	15,000	51	67	80	95	96	96	92	86	101
	12,000	52	68	81	96	96	96	93	87	102
	1000	39	55	73	78	83	83	80	74	88
Hold ventilation	95,000	75	93	97	100	100	97	91	83	105
	85,000	69	89	94	101	101	98	94	87	106
	73,000	67	83	99	102	104	102	97	91	109

**Table 6:** Comparison between data of EFFORT Project [18] and Tecnalia Report [27] related to the noise emission of some noise sources of Ro-Ro ships.

Ship Source	L_W_ (dB)	L_W_ (dB(A))
Engine room ventilation [18]	102	86
Hydraulic room ventilation [18]	110	104
Bow cable hydraulics [18]	103	93
Auxiliary engine exhaust pipe [18]	124	106
Exhaust stack ventilation [18]	112	100
Auxiliary engine [27]	/	107
Ventilation unit [27]	/	109

**Table 7:** Noise emission data of moored ship, as reported in [27]. * TEU: twenty-foot equivalent unit.

Type	Year	Size (GT)	Size (TEU *)	Reefer (TEU *)	Auxiliary Engine Power (kW)	Operating Conditions (kW)	Auxiliary Engine		Additional Source: Ventilation	
							L_w_ (dB(A)	Tonal Components/Low Frequency (dB)	Sound Power Level (dB(A))	Tonal Components/Low Frequency (dB)
Ro-Pax	2003	22,382	-	-	4200	900	109.3	6/0	113.2	6/0
Ro-Ro	1999	12,076	-	-	2 × 980	400	107.5	3/3	109.0	6/0
Containers	2002	14,241	1129	153	-	-	97.4	3/6	-	-
	2008	7702	798	150	2 × 750	1 × 750	95.1	0/3	-	-
	2007	8971	917	200	2 × 469	1 × 469	95.0	3/3	-	-
	2009	10,585	1036	-	-	-	90.2	0/3	92.3	3
Cruise ship	1973	28,372	-	-	2200	-	111.1	3/6	103.2	3/6
	2000	30,277	-	-	-	-	104.2	0/6	94.7	0/6
	2016	55,254	-	-	-	-	101.6	3/6	97.5	3/6
	2002	139,570	-	-	-	-	105.3	3/6	98.7	3/6
	2008	154,407	-	-	-	-	104.5	0/6	96.2	0/6

**Table 8:** Sound power level of Ro-Ro ramps, as reported in deliverable 2.4.3 of [18]. * The ship loading considers the whole measurement period, while other data consider the single event.

Ship Source	L_W_ (dB)	L_W_ (dB(A))
Ro-Ro ramp in Turku: tractor with trailer	114 ± 3	109 ± 3
Ro-Ro ramp in Turku: tractor without trailer	112 ± 4	106 ± 4
1st Ro-Ro ramp in Dublin: ramp noise	119 ± 5	112 ± 5
1st Ro-Ro ramp in Dublin: ship loading *	115 ± 2	108 ± 2
2nd Ro-Ro ramp in Dublin: ramp noise	121 ± 6	115 ± 6
2nd Ro-Ro ramp in Dublin: ship loading *	116 ± 2	109 ± 2
Ro-Ro ramp in Turku: tractor with trailer	114 ± 3	109 ± 3

**Table 9:** Sound power level of forklifts, as reported in [20].

Source Description	L_W_ (dB(A))	Data Taken from
Small/medium diesel forklift	97	Manufacturer
Heavy duty size diesel forklift	107	Manufacturer
Electric forklift	90	[42]
Mobile crane for cruise ship	107	[43]

**Table 10:** A-weighted SEL of seagoing ships, as reported in [48].

Ship Type	Direction	SEL (dB(A))
A	Arriving	87–90
A	Leaving	89–90
B	Arriving	88–91
B	Leaving	88–89

**Table 11:** One-third octave sound power spectra and sound power level of seagoing ships, as reported in [11,29,50]. Uncertainties can be retrieved from the original documents.

		One-Third Octave Frequency Bands (dB(A))																														
Ship Type	L_W_ dB(A)	20 Hz	25 Hz	31.5 Hz	40 Hz	50 Hz	63 Hz	80 Hz	100 Hz	125 Hz	160 Hz	200 Hz	250 Hz	315 Hz	400 Hz	500 Hz	630 Hz	800 Hz	1 kHz	1.25 kHz	1.6 kHz	2 kHz	2.5 kHz	3.15 kHz	4 kHz	5 kHz	6.3 kHz	8 kHz	10 kHz	12.5 kHz	16 kHz	20 kHz
Ro-Ro	87.5	99.2	98.3	96.7	97.7	96.9	97.1	94.8	91.4	90.0	88.8	86.2	84.6	83.1	81.2	80.1	78.7	77.2	75.9	74.1	72.5	70.9	68.8	67.1	65.6	64.8	64.5	64.4	67.0	68.6	69.2	69.2
Container	89.0	92.7	91.7	93.5	97.9	98.1	96.7	93.6	92.3	89.5	88.5	87.3	85.1	83.8	83.8	82.5	81.4	79.8	78.1	76.2	74.3	72.3	70.7	69.1	67.6	65.5	64.7	64.3	67.4	67.8	67.7	67.0
Oil tanker	82.6	86.5	85.9	88.3	90.5	89.0	87.3	89.5	87.6	84.2	80.3	78.5	78.0	76.7	75.1	74.2	73.1	72.4	71.5	70.9	69.3	67.8	68.6	66.6	64.7	64.0	64.4	63.6	65.0	66.9	67.6	67.5
Chemical tanker	85.9	87.1	89.5	90.6	94.4	95..0	91.8	91.1	89.2	88.3	84.8	83.2	84.5	81.3	79.8	78.6	77.3	75.9	75.4	73.1	71.8	69.6	67.8	67.0	65.0	63.7	62.6	62.1	64.1	65.3	66.1	67.5
Ferry	83.1	92.3	93.6	95.5	97.7	95.0	92.3	87.4	87.6	83.3	81.2	79.1	77.3	75.9	76.3	76.4	73.2	74.3	73.0	72.5	70.5	68.7	66.1	64.2	62.4	61.5	59.9	59.5	61.3	62.4	62.4	62.8
Small vessels	77.4	77.6	78.7	78.8	81.2	85.2	80.6	78.6	80.4	76.8	76.3	73.5	72.7	70.5	69.5	69.0	68.1	67.4	66.9	66.4	65.6	64.4	63.3	62.1	61.0	59.6	57.8	57.0	57.5	56.7	54.0	49.2
Medium vessels	83.5	78.1	80.4	85.5	86.3	90.9	85.6	87.3	89.7	87.4	84.4	82.1	80.9	77.4	75.5	75.4	75.1	73.8	72.6	70.9	69.6	68.0	66.9	65.8	64.2	62.3	60.4	59.8	60.3	59.6	56.6	51.7

## Data Availability

Not applicable.

## Appendix A

**Table A1:** Standards used in the acoustic measurements carried out to define the sound power level of port noise sources.

Reference	Port Noise Source	Measurement Carried out in Compliance with
REPORT Project [16]	Transtainer (Section 3.1)	NA
	Reach stacker (Section 3.2)	NA
	Gantry cranes (Section 3.4)	NA
	Reefer (Section 3.5)	NA
EFFORTS Project [17,18]	Transtainer (Section 3.1)	ISO 3744 *
	Reach stacker (Section 3.2)	ISO 3744 *
	Straddle carrier (Section 3.3)	ISO 3744 *
	Gantry cranes (Section 3.4)	ISO 3744 *
	Reefer (Section 3.5)	ISO 3744 *
	Moored ship (Section 3.6)	ISO 3744 *
	Ro-Ro and Ro-Pax ramps (Section 3.7)	ISO 3744 *
Witte, J. [19,22]	Straddle carrier (Section 3.3)	NA
	Gantry cranes (Section 3.4)	NA
	Moored ships	NA
Danish Ministry of the Environment [25]	Reefer (Section 3.5)	NA
	Moored ships (Section 3.6)	NA
GREEN CRUISE PORT [20]	Reefer (Section 3.5)	ISO 3746
	Engine of moored ships (Table 4 of Section 3.6)	ISO 9614-2
	Ventilation of moored ships (Table 5 of Section 3.6)	NA **
	Funnels of moored ships (Figure 8 of Section 3.6)	DIN 45 635-47:1985
	Ventilation openings of moored ships (Figure 8 of Section 3.6)	ISO 3746
	Diesel forklift (Section 3.7)	NA **
	Electric forklift (Section 3.7)	NA
NEPTUNES Project [21]	Reefer (Section 3.5)	NA
	Moored ships (Section 3.6)	NA
Tecnalia, [27]	Engine of Moored ships (Section 3.6)	ISO 3746 *
	Ventilation of Moored ships (Section 3.6)	ISO 3746 *
Di Bella and Remigi [24]	Moored ships (Section 3.6)	ISO 8297, ISO 3744 and ISO 3746
Moro [40]	Moored ships (Section 3.6)	ISO 3744
Di Bella et al. [48]	Seagoing ships (Section 3.9)	UNI 11143
Fredianelli et al. [29]	Seagoing ships (Section 3.9)	BS EN ISO 2922:2000 + A1:2013

* The authors declare that measurements were carried out via a simplified application of the standard. ** Data directly supplied by manufacturer.
